# Intimate partner violence in lesbian, gay, bisexual, transgender, queer, and intersex relationships: a call for research-to-action partnerships in higher education settings

**DOI:** 10.1186/s44263-024-00085-y

**Published:** 2024-08-02

**Authors:** Lu Gram, John Blevins, Stephanie Miedema, Anh Tu Hoang, Kathryn M. Yount

**Affiliations:** 1 Institute for Global Health, University College London, London, UK; 2 Hubert Department of Global Health, Emory University, Atlanta, GA USA; 3 Department of Sociology, Emory University, Atlanta, GA USA; 4 Center for Creative Initiatives in Health and Population, Hanoi, Vietnam

Intimate partner violence (IPV) in lesbian, gay, bisexual, transgender, queer, and intersex (LGBTQI +) relationships remains under-researched, particularly in the Global South. We call on research-to-action partnerships in higher education settings to address IPV in LGBTQI + relationships in partnership with queer communities as leaders in conceptualizing, designing, and conducting research activities.

## Intimate partner violence in LGBTQI + relationships

Despite substantial growth in scholarship on intimate partner violence (IPV) in lesbian, gay, bisexual, transgender, queer, and intersex (LGBTQI +) relationships, this research remains nascent. Scant existing evidence from gender-diverse LGBTQI + populations suggests high levels of IPV, requiring an urgent response. For example, data from the United States National Intimate Partner and Sexual Violence Survey 2016–2017 documented lifetime prevalence rates of IPV amongst self-identified sexual minority adults (bisexual women 69%; lesbian women 56%; bisexual men 46%; gay men 47%) at similar or higher rates than heterosexual adults (heterosexual women 46%; heterosexual men 44%) [1]. These adverse experiences exacerbate consistently elevated risks of poor mental health and suicide in LGBTQI + populations relative to their cis-heterosexual peers worldwide [2].

The evidence on causes of IPV in LGBTQI + relationships still is evolving. However, minority stress—stress derived from membership in stigmatized or marginalized minority groups—is known to be an intensifying factor for IPV amongst LGBTQI + people [3]. LGBTQI + people face stigma globally, ranging from subtle, yet pervasive forms of exclusion and marginalization to extreme acts of violence, and LGBTQI + people are at high risk of public physical or sexual violence due to their LGBTQI + status [4]. Internalized homophobia—self-stigmatization amongst LGBTQI + people due to homophobic attitudes and biases in the larger society—is associated with the perpetration of IPV [3]. Experiences of stigma and discrimination when interacting with police or health service providers, and fear of having to disclose their identity, can cause LGBTQI + people to avoid seeking recourse in response to experiences of IPV [3].

### Evidence gaps in the Global South

Evidence gaps on the extent, causes of, and solutions to IPV in LGBTQI + populations are most obvious in the Global South. Few rigorous studies on IPV in LGBTQI + populations exist in Global South contexts, and past funding, research, and advocacy have overwhelmingly focused on HIV/AIDS. For instance, out of 52 studies on the prevalence of IPV amongst men who have sex with men identified in a global systematic review, 38 took place in the Global North (35 in the USA, 1 in the United Kingdom, 1 in Spain, 1 in Canada), whilst only 12 took place in the Global South (11 in China, 1 in South Africa), and 2 took place in multiple countries [5].

Yet, efforts to close this research gap are greatly needed, as LGBTQI + populations in the Global South continue to face structural and interpersonal stigma and discrimination. As of 2024, at least 67 countries around the world have national laws criminalizing same-sex relations between consenting adults. In many such contexts, intense stigma and discrimination block LGBTQI + people’s educational attainment. For example, a study in Vietnam found over 40% of transmen and over 71% of transwomen experience transphobic bullying and violence in school, causing them to drop out of education [6].

### Institutions of higher education as entry points for prevention, empowerment, and leadership

Institutions of higher education are potential entry points for research and action to prevent IPV among LGBTQI + persons. Early adulthood is a period of rapid human and social development, in which young people are learning about their sexual and gender identity. Universities play a powerful role in shaping public attitudes about sexual and gender diversity in which LGBTQI + affirming networks, such as student societies, often are embedded. Conversely, a lack of engagement with higher education may harm global LGBTQI + rights, as education systems can also be used to promote intolerance. A study of attitudes to same-sex relations in 88 countries found evidence for increasing tolerance with higher levels of education, except in countries with low political freedom, where higher education was associated with *lower* levels of tolerance [7].

Bystander programs engaging LGBTQI + people and allies in speaking out against IPV and homophobia and referring survivors of IPV to LGBTQI + friendly services could play a critical role. Bystander programs to prevent sexual and relationship violence have improved participants’ ability to identify and intervene in violence and reduced the perpetration of violence in countries as varied as Vietnam, China, India, the United Kingdom, and the USA [8, 9]. Yet, a systematic review of such programs in the USA noted that *none* of the 28 evaluations included in the review reported disaggregated impacts on LGBTQI + participants [10]. Only two studies explicitly stated a lack of LGBTQI + inclusion as a study limitation. These evidence gaps exist against the background of a general lack of evidence on the prevention of violence in university settings in the Global South: In a global systematic review, out of 24 evaluations of bystander programs to address IPV and sexual violence in university settings, only two took place in the Global South (India and China) [8], to which we are only aware of one other such evaluation, a sexual violence prevention program tailored to university men in Vietnam [9].

Programs to prevent relationship violence in university settings require adaptation to work for LGBTQI + students. Conceptualizations of IPV as an extension of cis-men’s patriarchal power over cis-women fit awkwardly with same-sex and queer relationships [3]. Intersectional perspectives that recognize the uniqueness of LGBTQI + experiences are needed, as IPV in LGBTQI + relationships can assume forms not seen in cis-heterosexual relationships, such as threats to “out” or disclose partners’ sexual minority status to family members or the public, use of derogatory terms to describe partners’ sexual and/or gender identity, or control over partners’ social life in the LGBTQI + community [3]. Equally needed are post-structuralist and queer theoretical perspectives that conceptualize gender and sexual identity as fluid, performative, and context-dependent and interpret attempts to control people’s sexual or gender presentation as a form of psychological violence [3].

### Time for action

It is time to foster new avenues in higher education to support young people who experience IPV in LGBTQI + communities and generate rigorous evidence to inform expanded action. New studies providing a nuanced, contextualized understanding of the full diversity of LGBTQI + identities, relationships, and lived experiences, as well as strategies for resilience would help to develop suitable theories for change. Culturally sensitive, locally relevant studies would contribute to the decolonization of LGBTQI + research, hitherto too often based on Northern epistemologies. Tentative signs of a shift are apparent in the priorities of development agencies and research funders. For example, as part of the United States Agency for International Development (USAID) strategy for LGBTQI + inclusive policy, USAID is integrating measures of LGBTQI + identity into routine Demographic and Health Surveys covering over 90 LMICs [11].

However, research on IPV in LGBTQI + relationships in the Global South must be done in genuine partnership with local queer communities and must account for the full diversity and fluidity of LGBTQI + identities and relationships, lest it becomes another form of intellectual neocolonialism, in which epistemic categories from the Global North are imposed on peoples of the Global South. Research should be done with members of local queer communities as leaders in conceptualizing, implementing, and conducting any activities, and should center the voices of local populations that are experiencing intersecting forms of oppression. In doing such research, we extend traditional feminist approaches to IPV prevention and response toward new paradigms that include people of diverse sexual orientations and gender identities from all regions of the world.

## Data Availability

No datasets were generated or analysed during the current study.
